# Diagnostic utility of procalcitonin in determining Gram-negative and Gram-positive bacterial bloodstream infections: a single-centre study from South India

**DOI:** 10.3389/fmed.2026.1762021

**Published:** 2026-04-01

**Authors:** Deepashree Rajashekar, Sujatha S. R., Vidyavathi B. Chitharagi, Yogeesh D. Maheshwarappa, Amrutha Varshini I., Veerabhadra Swamy G. S., Chinchana Shylaja Eshwarappa

**Affiliations:** Department of Microbiology, JSS Medical College and Hospital, JSS Academy of Higher Education and Research, Mysuru, India

**Keywords:** blood culture, bloodstream infections, ELFA, PCT values, procalcitonin

## Abstract

**Background:**

Bloodstream infections (BSIs) are significant contributors to global morbidity and mortality, with sepsis accounting for 11 million deaths annually. Accurate and rapid diagnosis is essential for effective management.

**Methods:**

This retrospective analysis was conducted on 275 positively flagged blood culture bottles collected over one year. Samples were processed using automated blood culture systems, followed by pathogen identification and antimicrobial susceptibility testing (AST) via the Vitek-2 Compact system (bioMérieux, France). PCT (Procalcitonin) levels were measured using MINIVIDAS (bioMérieux, France) and correlated with pathogens to evaluate diagnostic accuracy.

**Results:**

Among 251 isolates, Gram-negative bacteria predominated (77.68%), with Enterobacterials like *Escherichia coli* (46.3%) and *Klebsiella pneumoniae* (44.29%) being the most common. GP (Gram-positive) bacteria accounted for 22.31%, led by *Staphylococcus aureus* and *Enterococcus* species. The mean PCT levels were higher in GN (Gram-negative) infections (37.87 ng/mL) than in GP infections (35.09 ng/mL). PCT demonstrated a sensitivity of 77.41%, specificity of 91.04%, and diagnostic accuracy of 82.87%.

**Conclusion:**

PCT levels were elevated in both Gram-negative and Gram-positive BSIs, with marginally higher values in Gram-negative infections. Significant elevations in Gram-positive infections, particularly with *Streptococcus* and *Staphylococcus* species, highlight their broader diagnostic relevance. With good sensitivity and high specificity, PCT serves as a useful adjunctive biomarker for early BSI detection and may support preliminary differentiation of pathogen groups when interpreted alongside clinical and microbiological findings.

## Introduction

Bloodstream infections (BSIs) are defined by the presence of viable bacterial or fungal pathogens in the bloodstream that elicit a severe inflammatory response characterised by the alteration of clinical, laboratory and hemodynamic parameters (1). BSIs are one of the major worldwide health concerns that increase economic burden, morbidity, and death (2). These infections range from acute sepsis and septic shock to temporary bacteremia, all of which have serious systemic effects that need rapid diagnosis and treatment (3).

In 2017, there were 48.9 million cases of sepsis and 11 million deaths globally, accounting for one in five deaths worldwide, highlighting sepsis as a significant global health priority as per the WHO’s 2020 report (4). Additionally, the financial burden on healthcare systems is considerable due to prolonged hospital stays, intensive care unit (ICU) admissions, and the use of advanced therapeutic interventions (5). Sepsis is defined as life-threatening organ dysfunction caused by a dysregulated host immune response to infection (6). Septic shock is characterised by circulatory and metabolic abnormalities, leading to reductions in tissue perfusion and a heightened risk of death (7). Early recognition and appropriate management of BSIs are crucial, as delays in intervention can result in irreversible organ damage and death (8).

BSIs are caused by various pathogens, including bacteria, fungi, and, less frequently, viruses (9). The most prevalent bacterial infections include Gram-negative bacteria such as *Escherichia coli*, *Klebsiella pneumoniae*, *Pseudomonas aeruginosa*, and *Salmonella* species, and Gram-positive bacteria such as *Staphylococcus aureus* (including MRSA) and *Streptococcus pneumoniae* (10). Fungal infections, particularly those caused by *Candida* species, are increasingly determined in immunocompromised individuals (11). Additionally, patient location, demographics, and medical/surgical procedures all have a significant impact on the epidemiology of BSIs and the development of MDR organisms, which pose serious diagnostic and treatment challenges (12).

The diagnosis of bloodstream infections (BSIs) primarily depends on clinical suspicion, blood cultures, and other laboratory investigations. Blood culture remains the gold standard for identifying pathogens (13); however, it has certain limitations, such as a prolonged turnaround time of 24–72 h and low sensitivity in patients who have previously received antibiotics, potentially leading to false-negative results (14). In recent years, several alternative detection methods have been developed to complement conventional culture techniques. These methods can directly detect BSIs from positive blood cultures or whole blood and have improved the speed, sensitivity, and specificity of pathogen identification. They include polymerase chain reaction (PCR), fluorescence *in situ* hybridisation (FISH), matrix-assisted laser desorption/ionisation-time of flight mass spectrometry (MALDI-TOF-MS), biosensors, and microfluidic-based systems. Some of these rapid diagnostic tools have shown success in reducing the time required for appropriate therapy, lowering healthcare costs, and shortening hospital stays. Furthermore, they have been linked to a decreased mortality risk when used alongside antimicrobial susceptibility testing (AST) (15, 16).

Biomarkers play a crucial role in the diagnosis and management of sepsis, as they can indicate its presence, absence, or severity (17). They are also useful in distinguishing bacterial infections from viral and fungal infections, as well as systemic sepsis from localised infections. Additional potential applications of biomarkers include aiding in prognostication, guiding antibiotic therapy, monitoring response to treatment and recovery, distinguishing between Gram-positive and Gram-negative microorganisms as the cause of sepsis, predicting complications of sepsis, and assessing the risk of organ dysfunction (e.g., heart, kidney, liver, or multiple organ dysfunction). However, the precise role of biomarkers in the management of septic patients remains uncertain (18).

C-reactive protein (CRP) has been widely used for many years (19), though its specificity has been questioned (19). Procalcitonin (PCT) has been suggested as a more specific and superior prognostic marker compared to CRP, yet its utility is also subject to debate (20). Differentiating sepsis from other non-infectious causes of systemic inflammatory response syndrome remains challenging, driving the ongoing search for more reliable sepsis biomarkers. The present study aimed to evaluate the diagnostic utility of procalcitonin in culture-proven bloodstream infections and to compare PCT levels between Gram-positive and Gram-negative bacterial infections. Although previous studies have evaluated procalcitonin in bloodstream infections, data from South Indian tertiary-care settings with a high Gram-negative and multidrug-resistant burden remain limited. The present study adds pathogen-specific and region-relevant evidence on the diagnostic performance of PCT under routine clinical conditions.

## Methodology

### Sample collection

Clinical samples from suspected cases received at the Microbiology Laboratory of JSS Hospital, Mysuru, South India, over one year, served as the source sample for this study. All patients included in this study were recruited from inpatient departments (IPD) of our tertiary care hospital, and no outpatients were included. The paired blood (right and left hand) samples were collected aseptically using 2% chlorohexidine and 2% tincture of iodine for site preparation. Using sterile syringes, 7–10 mL of blood was drawn from adults and 3–5 mL from paediatric patients. These samples were promptly transferred to blood culture bottles (bioMérieux, France). Additionally, 3 mL blood samples were transferred into EDTA sample collection tubes for Widal and the immunochromatographic test. The demographic details of the patients were collected from case sheets and the Hospital information system, and before conducting the study authors obtained JSS Medical College and Hospital institutional ethical committee approval.

### Blood culture

Blood culture was performed using an automated blood culture machine, BACT/ALERT 3D (bioMérieux, France). The positive-flagged blood culture bottles were subjected to microscopic examination (Gram’s staining), subcultured on suitable media (Blood Agar and MacConkey Agar), and incubated at appropriate conditions (37 °C for 24 h, aerobically).

### Microscopic examination

The positively flagged blood culture bottles were subjected to Gram staining. Any presence of Gram-positive cocci in paired blood culture bottles collected from different sites was considered significant, and the same was informed to the physician as a critical alert. Similarly, the presence of Gram-negative bacilli in any one of the positively flagged blood culture bottles is considered significant and communicated to the physician as a critical alert. In children, Gram-positive cocci are isolated, where they proceed for identification and antimicrobial susceptibility testing (Vitek-2 compact system by bioMérieux, France) with a note to clinicians to rule out the possibility of contamination.

### Pathogen identification and antimicrobial susceptibility testing

The suspected pathogens were identified using conventional biochemical tests and an automated identification system (Vitek-2 compact system by bioMérieux, France). The AST was performed using the Vitek-2 Compact system. The results of AST were interpreted according to the CLSI guidelines as susceptible, intermediate and resistant. To check the quality of the results, internal quality control (IQC) tests were conducted on the Vitek-2 Compact system once a week by using Gram-positive (ATCC 25922 *Staphylococcus aureus*, ATCC 29212 *Enterococcus faecalis* and ATCC 25788 *Enterococcus casseliflavus*) and Gram-negative (ATCC 25923 *Escherichia coli*, ATCC 27853 *Pseudomonas aeruginosa*, ATCC 17666 *Stenotrophomonas maltophilia*) and ATCC *Candida albicans* strains.

## Results

### General demographic details

In this retrospective study, the authors collected 275 positively flagged paired blood culture bottles of bacteremia patients, of which 251 bottles yielded defined pathogenic bacteria. Among the 251 bottles, male patients predominated with 57.76% (145/251), followed by females at 42.23% (106/251). Additionally, most of the positive blood culture bottles are from the 61–89 age group, accounting for 39.44% (99/251), followed by the 41–60 age group, accounting for 31.47% (79/251), and the least represented age group was 1–20, accounting for 2.39% (6/251). Additionally, the majority of patients with positively flagged bottles were diagnosed with sepsis/septic shock (27.4%, 69/251), followed by respiratory tract infections (23.5%, 59/251) and urogenital infections (21.91%, 55/251). The least common diagnoses were CNS infections (6.3%, 16/251) and skin/soft tissue infections (6.7%, 17/251).

### Associated WBC count in patients

The majority of patients with bacteremia had elevated WBC counts (>11,000), accounting for 60% (151/251). Additionally, 32 patients had WBC levels <4,000, and the remaining 68 patients had WBC values ranging from 4,000 to 11,000.

### Pathogen distribution

The majority of the causative agents were Gram-negative organisms, accounting for 77.68% (195/251). In 195 Gram-negative organisms, Enterobacterials accounted for 76.41% (149/195), followed by non-fermenters at 23.58% (46/195). The predominant Enterobacterials were *Escherichia coli* and *Klebsiella pneumoniae*, accounting for 46.30% (69/149) and 44.29% (66/149), respectively. The Gram-positive organisms accounted for 22.31% (56/251), with *Staphylococcus* and *Enterococcus* species predominating, accounting for 37.5% (21/56) and 32.1% (18/56), respectively.

### Antimicrobial susceptibility patterns

#### Susceptibility patterns of Gram-positive organisms

Among the 17 *Staphylococcus aureus* isolates, varying susceptibility patterns were observed across the different antimicrobial agents. A high level of resistance was observed to oxacillin (66%), ciprofloxacin (72.2%), and levofloxacin (72.2%). Even cotrimoxazole showed particularly poor activity, with 88.9% of isolates resistant. In contrast, higher susceptibility rates were observed for teicoplanin (94.4%) and gentamicin (88.2%). Daptomycin and tetracycline demonstrated similar susceptibility, with 77.7% of isolates sensitive to each. Vancomycin retained activity against 66.6% of isolates.

*Streptococcus* species showed high resistance rates to ciprofloxacin and daptomycin. Trimethoprim/sulfamethoxazole also demonstrated poor activity, with 88.9% resistance. Penicillin resistance was noted in 62% of isolates. Higher susceptibility was observed to vancomycin (76.1%) and teicoplanin (71.4%). Tetracycline and levofloxacin showed sensitivity rates of 61.9 and 57.1%, respectively. Among the *Enterococcus* species, high resistance rates were observed to ciprofloxacin (83.4%), levofloxacin (77.8%), gentamicin (77.8%), daptomycin (88.9%), and tetracycline (72.3%). Penicillin demonstrated low activity, with only 22.2% of isolates sensitive. Higher susceptibility was noted for teicoplanin (83.3%), followed by vancomycin (see Table 1).

**Table 1:** Antimicrobial susceptibility patterns of *Staphylococcus aureus*, *Streptococcus* species, and *Enterococcus* species.

Antimicrobial agent	*Staphylococcus aureus* (17)		*Streptococcus* species (21)		*Enterococcus* species (18)	
	Sensitive	Resistant	Sensitive	Resistant	Sensitive	Resistant
Penicillin	—	—	38.0%	62.0%	22.20%	77.80%
Oxacillin	44%	66%	—	—	—	—
Gentamicin	88.20%	11.80%	—	—	22.20%	77.8%
Trimethoprim/Sulfamethoxazole	11.10%	88.90%	11.10%	88.90%	—	—
Ciprofloxacin	27.70%	72.20%	4.70%	95.3%	16.60%	83.40%
Levofloxacin	27.70%	72.20%	57.10%	42.90%	22.20%	77.80%
Daptomycin	77.70%	22.30%	4.70%	95.3%	11.10%	88.90%
Tetracycline	77.70%	22.30%	61.90%	38.10%	27.70%	72.30%
Tigecycline	50%	50%	38.90%	61.10%	72.20%	27.80%
Vancomycin	66.60%	33.40%	76.10%	23.90%	72.20%	27.80%
Teicoplanin	94.40%	5.60%	71.40%	28.60%	83.30%	16.70%

#### Susceptibility patterns of Gram-negative organisms

The antimicrobial susceptibility profile of *E. coli* and *K. pneumoniae* demonstrated considerable resistance across different antibiotic classes. Among *E. coli*, the highest resistance rates were observed for cefuroxime and cefuroxime axetil (62% each), followed by cefepime (59%), trimethoprim/sulfamethoxazole (56.82%), fluoroquinolones (51.66%), and cefoperazone/sulbactam (51.90%). Resistance to aminoglycosides and ceftriaxone was 50.85 and 50%, respectively, while comparatively lower resistance was seen with amoxicillin/clavulanate (48.39%) and carbapenems (41.70%).

*K. pneumoniae* exhibited a higher resistance pattern compared to *E. coli*. The higher resistance was noted for cefepime (64.52%), cefuroxime and cefuroxime axetil (62.96% each), and trimethoprim/sulfamethoxazole (60.40%). Resistance to fluoroquinolones and cefoperazone/sulbactam was 54.84% each, followed by ceftriaxone (54.84%), aminoglycosides (51.85%), and carbapenems (51.80%). The lowest resistance among *K. pneumoniae* isolates was observed for amoxicillin/clavulanate (48.39%) (see Table 2).

**Table 2:** Antimicrobial susceptibility patterns of *Escherichia coli* and *Klebsiella pneumoniae* species.

Antimicrobial agent	*Escherichia coli* (69)		*Klebsiella pneumoniae* (66)	
	Sensitive	Resistant	Sensitive	Resistant
Carbapenems	58.30%	41.70%	48.20%	51.80%
Fluroquinolones	48.34%	51.66%	45.16%	54.84%
Aminoglycosides	49.15%	50.85%	48.15%	51.85%
Amoxicillin/Clavulanate	51.61%	48.39%	51.61%	48.39%
Cefoparazone/Sulbactam	48.10%	51.90%	45.16%	54.84%
Trimethoprim/Sulfamethoxazole	43.18%	56.82%	39.60%	60.40%
Cefepime	41.00%	59.00%	35.48%	64.52%
Ceftriaxone	50.00%	50.00%	45.16%	54.84%
Cefuroxime	38.00%	62.00%	37.04%	62.96%
Cefuroxime axetil	38.00%	62.00%	37.04%	62.96%

#### PCT values associated with Gram-positive organisms

Table 3 depicts the mean, median, and range of PCT values of Gram-positive organisms. The mean PCT value was found to be highest in *Streptococcus aureus* (53.07 ng/mL), followed by *Staphylococcus aureus* (32.98 ng/mL) and *Enterococcus* species (19.22 ng/mL).

**Table 3:** PCT values associated with Gram-positive organisms.

Isolate name	Number of isolates	Percentage of isolates	Mean PCT in ng/mL	Median PCT in ng/mL	PCT range in ng/mL
*Staphylococcus aureus*	17	6.7	32.98	2.2	(200–0.15) 199.85
*Enterococcus* species	18	7.17	19.22	7.08	(200–0.07) 199.93
*Streptococcus* species	21	8.4	53.07	22.14	(200–0.85) 199.85

The percentage of the isolates was calculated by dividing the total no of isolates.

#### PCT values associated with Gram-negative organisms

The mean PCT value of Gram-negative organisms was similar to that of Gram-positive organisms, at 30.84 ng/mL. The average PCT value was found to be higher in the non-fermenter group, particularly for *Pseudomonas aeruginosa* (64.09 ng/mL), followed by *E. coli* (57.92 ng/mL) and *Klebsiella pneumoniae* (50.50 ng/mL) from the Enterobacterials as depicted in Table 4.

**Table 4:** PCT values associated with Gram-negative organisms.

Isolate name	Number of isolates	Percentage of isolates	Mean PCT in ng/mL	Median PCT in ng/mL	PCT range in ng/mL
Enterobacterials					
*Escherichia coli*	69	27.4	57.92	43.63	(2000–0.06) 199.94
*Klebsiella pneumoniae*	66	26.2	50.50	28.18	(200–0.05) 199.95
Other Enterobacterials	14	5.57	13.6	3.37	(17.61–0.11) 17.5
Non-fermenters					
*Acinetobacter baumanii*	29	11.55	38.54	3.5	(200–0.05) 199.95
*Pseudomonas aeruginosa*	14	5.57	64.09	24.78	(200–0.09) 199.19
Other non-fermenters	3	1.1	2.57	2.82	(4.12–0.77) 3.35

#### Diagnostic accuracy of procalcitonin

True positives, true negatives, false positives, and false negatives were assessed using the interpretation table provided in the bioMérieux procalcitonin handbook. Out of 251 total pathogens isolated, 233 bottles were true positives (blood culture positive + PCT values positive), while the remaining 18 bottles were true negatives (blood culture positive + PCT values negative). The study found that PCT had a sensitivity of 77.41% (CI range 72.26–82%), specificity of 91.04% (CI range 86.22–94.61%), PPV of 92.83%, NPV of 72.81%, and a diagnostic accuracy of 82.87% (see Table 5).

**Table 5:** Diagnostic accuracy of procalcitonin.

Parameters	Values	95% CI
Sensitivity	77.41%	72.26 to 82%
Specificity	91.04%	86.22 to 94.61%
Prevalence	59.96%	55.53 to 64.28%
Positive predictive value	92.83%	89.24 to 95.28%
Negative predictive value	72.91%	68.49 to 76.92%
Diagnostic accuracy	82.87%	79.28 to 86.06%

CI, confidence intervals.

## Discussion

This study analysed 251 positively flagged paired blood culture bottles, predominantly from male patients (57.76%), with the majority of isolates obtained from the elderly population (61–89 years, 39.44%). These findings align with previous studies indicating that elderly patients, especially males, are more susceptible to bloodstream infections (BSIs) due to immunosenescence, comorbidities, and prolonged hospitalisations (21–23).

The most common clinical diagnosis associated with positive blood cultures was sepsis or septic shock (27.4%), followed by respiratory tract infections (23.5%) and urogenital infections (21.91%). This distribution highlights the significant role of BSIs in severe systemic infections, often secondary to primary infections in the respiratory and genitourinary tracts (24–26). Less frequent diagnoses included central nervous system (CNS) infections (6.3%) and skin and soft tissue infections (6.7%), consistent with previous studies indicating their lower propensity to cause bacteremia (10, 26).

Culture results revealed a predominance of Gram-negative bacteria (GNB) of 77.68% of the isolates. Among the Enterobacterials, including *E. coli* (46.3%) and *K. pneumoniae* (44.29%) were the most common pathogens. Non-fermenters comprised 23.58% of the isolates, with *P. aeruginosa* (5.57%) and *A. baumannii* (11.55%) being major contributors. These findings reflect the increasing role of GNB in BSIs, particularly in hospitalised and critically ill patients (10, 27–30). In contrast to our study, other studies have reported a predominance of Gram-positive bacteria (GPB) in BSIs, reflecting geographical and institutional variability (31–33). In our study, GPB accounted for 22.31% of isolates, with *Staphylococcus aureus* (6.7%) and *Enterococcus* species (7.17%) being major pathogens (32).

In our study, PCT levels were determined as a diagnostic marker for distinguishing between Gram-negative (GN) and Gram-positive (GP) bacterial infections in patients with BSIs, with the observed differences in PCT values attributed to the distinct immune response pathways triggered by these pathogens (34). In our study, the mean PCT value in Gram-negative infections (37.87 ng/mL) was slightly higher compared to Gram-positive infections (35.09 ng/mL). Similar findings were reported in studies conducted by Bilgili et al. (34) and Thomas-Rüddel et al. (35), which demonstrated higher PCT levels in Gram-negative BSIs compared to Gram-positive BSIs. Gram-negative bacilli activate Toll-like receptor 4 (TLR4) via lipopolysaccharide (LPS), leading to activation of an inflammatory cascade involving proinflammatory cytokines such as tumour necrosis factor-alpha (TNF-α), interleukin (IL)-1, IL-6, IL-8, and IL-10, which stimulates PCT synthesis (34, 36). Elevated PCT levels in Gram-negative infections may also reflect their association with severe infections and higher mortality rates (37).

The mean PCT value in Gram-positive infections was lower but remained significant, with *Streptococcus* species showing the highest mean PCT value (53.07 ng/mL), followed by *Staphylococcus aureus* (32.98 ng/mL). Similarly, a study conducted by Koizumi et al. (38) reported a significantly lower median PCT level (0.50 ng/mL) in cases of Gram-positive bacteremia. The relatively lower PCT levels in Gram-positive infections may be due to differences in the inflammatory response triggered by Gram-positive pathogens, which activate Toll-like receptor 2 (TLR2) through lipoteichoic acid, eliciting a comparatively weaker cytokine response (34, 38–41).

The diagnostic accuracy of PCT in determining bloodstream infections was notable, with a sensitivity of 77.41%, specificity of 91.04%, and an overall diagnostic accuracy of 82.87%. The high positive predictive value (PPV) of 92.83% highlights the utility of PCT as a reliable marker of bloodstream infection. However, the negative predictive value (NPV) of 72.81% suggests that a negative PCT result should be interpreted cautiously alongside clinical findings and other laboratory markers. Similar findings were reported by a study conducted by Shokripour et al. (42) and Habib et al. (43), with slight variations attributed to differences in study setting and patient populations. Shokripour et al. (42) reported comparable findings, with a sensitivity of 75%, an NPV of 91.5%, and a specificity of 79.9%. Similarly, a study by Habib et al. (43) reported a sensitivity of 97.7%, specificity of 70.6%, PPV of 77.1%, and NPV of 96.8%.

A study conducted by Zamyatina and Heine (36) concluded that PCT thresholds for differentiating GN from GP infections varied widely, reflecting heterogeneity in study designs and methodologies. Despite these variations, the consistent observation of higher PCT levels in Gram-negative infections highlights the reliability of PCT as a diagnostic marker for distinguishing between bacterial pathogens in sepsis. Notably, PCT levels also correlated with disease severity, with higher levels observed in Gram-negative bacteremia, often associated with critically ill patients and worse clinical outcomes (44–47). This underscores the potential utility of PCT not only as a diagnostic marker but also as a prognostic indicator in sepsis management.

### Limitations

- This is a single-centre retrospective study, which may limit the generalizability of the findings to other institutions and geographic regions.
- This study does not classify the bloodstream infections as hospital-acquired (HA-BSIs) or community-acquired (CA-BSIs). Since epidemiology, pathogen profile, and resistance patterns differ between these groups, the lack of stratification may limit clinical interpretability.
- This study evaluated the PCT levels at a single time; serial measurements that could better reflect disease progression and response to therapy were not assessed.
- Additionally, clinical severity scores (e.g., SOFA/qSOFA) and patient outcomes (mortality, length of stay, ICU admission) were not systematically correlated with PCT levels.

## Conclusion

Bloodstream infections remain a significant global health burden, particularly among hospitalised elderly patients. Gram-negative bacteria, such as *E. coli*, and *K. pneumoniae*, were the leading pathogens in BSIs. Procalcitonin (PCT) proved to be a reliable biomarker for BSIs, though its interpretation should complement clinical and microbiological findings. The rising prevalence of multidrug-resistant organisms highlights the need for robust antimicrobial stewardship strategies, continuous surveillance, and advanced diagnostic methods to improve outcomes and reduce healthcare costs. Early identification of pathogens and tailored therapy are critical in minimising morbidity and mortality, and the economic impact of BSIs. Focused studies on regional antimicrobial resistance patterns and biomarker utility will further aid in optimising patient care.

## Data Availability

The original contributions presented in the study are included in the article/supplementary material, further inquiries can be directed to the corresponding author.
